# MALAT1 functions as a competing endogenous RNA to regulate SMAD5 expression by acting as a sponge for miR-142-3p in hepatocellular carcinoma

**DOI:** 10.1186/s13578-019-0299-6

**Published:** 2019-05-10

**Authors:** Qiangfeng Yu, Leyang Xiang, Zhanjun Chen, Xincheng Liu, Huohui Ou, Jianyin Zhou, Dinghua Yang

**Affiliations:** 1 Department of Hepatobiliary Surgery, The Second Hospital of Longyan, Fujian, China; 2 Department of Hepatobiliary Surgery, Nanfang Hospital Affiliated to Southern Medical University, Guangzhou, China; 3 The Second Affiliated Hospital of Shantou University Medical College, Shantou, China; 4 Department of Hepatobiliary and Pancreatic Surgery, Zhongshan Hospital, Xiamen University, Xiamen, China

**Keywords:** MALAT1, miR-142-3p, Hepatocellular carcinoma, EMT, SMAD

## Abstract

**Background:**

Long non-coding RNAs are involved in the pathology of various tumors, including hepatocellular carcinoma. The expression of metastasis-associated lung adenocarcinoma transcript 1 (MALAT1) is increased in numerous types of tumors and is involved in tumor cell proliferation, migration, invasion and apoptosis. MALAT1 level was reported to be upregulated in hepatocellular carcinoma tissues, but its roles and the specific molecular mechanisms are still unclear.

**Methods:**

The expression of MALAT1 and miR-142-3p in hepatocellular carcinoma tissues, cell lines and adjacent non-tumor tissues was assessed by Q-PCR. The putative-binding sites between MALAT1 and miR-142-3p were predicted by bioinformatics analysis. The expression of MALAT1 in HepG2 and SMMC-7721 cells was knocked down by transfection with MALAT1 siRNAs. Cell viability was assessed by the Cell Counting Kit-8 (CCK-8) assay after the indicated transfection in HepG2 and SMMC-7721 cells. Cell proliferation was assessed by EdU assay, and cell apoptosis was explored by flow cytometry. The migration and invasion potency of HepG2 and SMMC-7721 cells was assessed by the cell migration assay and matrigel invasion assay. Protein level of vimentin, E-cadherin and SMAD5 were assessed by Western blot.

**Results:**

Overexpressed MALAT1 acts as a competing endogenous RNA sponge for miR-142-3p in hepatocellular carcinoma. The knockdown of MALAT1 inhibited the proliferation, migration, invasion, and epithelial cell-to-mesenchymal transition (EMT), and promoted apoptosis of hepatocellular carcinoma cells via miR-142-3p. MiR-142-3p inhibited cell proliferation, migration, invasion and EMT, and promoted the cell apoptosis by targeting SMAD5 in hepatocellular carcinoma. MALAT1 promoted tumor growth by regulating the expression of miR-142-3p in vivo.

**Conclusion:**

MALAT1 promoted cell proliferation, migration, and invasion of hepatocellular carcinoma cells by antagonizing miR-142-3p.

## Introduction

As the second leading cause of cancer-related deaths worldwide, hepatocellular carcinoma has led to approximately 800,000 deaths and is associated with 850,000 new cases each year [1]. Hepatitis B and C viral infection, intake of alcohol, and exposure to the fungal metabolite aflatoxin B1 are the main risk factors for hepatocellular carcinoma [2, 3]. Primary hepatocellular cancer is a type of heterogeneous tumor in which the tumors metastasizes to hepatocellular tissue from other sites. As the main primary hepatocellular cancer, hepatocellular carcinogenesis is multifactorial process with different susceptibility factors. Large regional variations in the prevalence of hepatocellular carcinogenesis have been reported [4, 5]. As the most common primary liver cancer, hepatocellular carcinoma is heterogeneous in nature [6]. Many molecular and cell signaling pathways have been implicated in the development of hepatocellular carcinoma, and the progress of tumorigenesis in hepatocellular carcinoma remains unclear. Most important in the effort to improve survival in patients with hepatocellular carcinoma patients is the identification of therapeutic targets for the disease.

Long noncoding RNAs (lncRNAs) are a heterogeneous class of transcripts longer than 200 nucleotides with limited protein-coding ability but play an important role in the pathogenesis of hepatocellular carcinoma [7]. Some lncRNAs are aberrantly expressed in hepatocellular carcinoma or other human cancers where their interaction with various macromolecules such as DNA, chromatin, proteins, and RNAs affects cell proliferation, apoptosis, angiogenesis, invasion, and metastasis of hepatocellular carcinoma cells [8–10]. The aberrantly expressed lncRNAs in hepatocellular carcinoma may potentially prove useful as prognostic or diagnostic biomarkers [11–15]. Because of the important roles of MALAT1 in many diseases, especially cancer, MALAT1 is receiving more and more attention. It was confirmed that the expression of MALAT1 was upregulated in various types of tumors and that MALAT1 has a significant influence on tumor cell proliferation, migration, invasion, and apoptosis [16–20]. The upregulated expression of MALAT1 was found in almost in every organ of the digestive system including hepatocellular carcinoma tissue, but its roles and the specific molecular mechanisms are still unclear.

In our study, we confirmed that MALAT1 was upregulated in hepatocellular carcinoma tissues compared with adjacent non-tumor tissues. We found that MALAT1 acted as a miRNA decoy for miR-142-3p and regulated the expression of miR-142-3p in hepatocellular carcinoma cells. The knockdown of MALAT1 inhibited proliferation, migration, invasion and EMT, and promoted cell apoptosis of hepatocellular carcinoma cells via miR-142-3p. MiR-142-3p suppressed cell proliferation migration, invasion and EMT, and promoted cell apoptosis by targeting SMAD5 in hepatocellular carcinoma. In the established xenograft model, MALAT1 promoted tumor growth by regulating the expression of miR-142-3p. Taken together, our results suggest that MALAT1 promoted cell proliferation, migration, and invasion by acting as a miRNA decoy for miR-142-3p.

## Methods

### Cell lines

The cell lines Bel-7402, Huh-7, HepG2 and SMMC-7721 were obtained from the American Type Cell Culture (Manassas, VA). Human liver cell line HL-7702 was bought from the Shanghai Institute for Biological Sciences, Chinese Academy of Sciences. HL-7702 and Bel-7402 cells were cultured in RPMI-1640 containing 1% penicillin and streptomycin. The Huh-7, HepG2 and SMMC7721 cell lines were cultured in Dulbecco’s Modified Eagle Media (DMEM). Both the RPMI-1640 and DMEM medium were supplemented with 10% fetal bovine serum (FBS) and cultured in a humidified incubator under 5% CO_2_ at 37 °C.

### Clinical samples

The hepatocellular carcinoma tissues and adjacent non-tumor tissues were collected from hepatocellular carcinoma patients during surgery at Nanfang Hospital Affiliated to Southern Medical University. Informed consent was obtained from all patients and the study protocol was approved by the institutional ethics committee of Nanfang Hospital Affiliated to Southern Medical University. The tumor tissues were immediately frozen and kept at − 80 °C.

### RNA isolation and quantitative real-time PCR (Q-PCR)

Trizol reagent (Invitrogen, Carlsbad, CA) was used to extract the total RNA from hepatocellular carcinoma tissues and cells. Random primers, 1 μg RNA template and Primescript reverse transcriptase (Takara, Japan) were used for single strand cDNA synthesis. Q-PCR was performed for MALAT1, glyceraldehyde 3-phosphate dehydrogenase (GAPDH), SMAD5, miR-142-3p and U6. Primer sequences were: MALAT1, Forward, 5′-ACGATGGTGTCGAGGTCTTT-3′ and reverse, 5′-TCCCACCCAGCATTACAGTT-3′; GAPDH, Forward, 5′-TGTTCGTCATGGGTGTGAAC-3′ and reverse, 5′-ATGGCATGGACTGTGGTCAT-3′; SMAD5, Forward, 5′- CCAGCAGTAAAGCGATTGTTGG-3′ and reverse, 5′-GGGGTAAGCCTTTTCTGTGAG-3′; miR-142-3p, Forward, 5′-ACACTCCAGCTGGGTGTAGTGTTTCCTACTTTA-3′, and reverse, 5′-CTCAACTGGTGTCGTGGA′; U6, Forward, 5′-CTCGCTTCGGCAGCACA-3′ and reverse, 5′-AACGCTTCACGAATTTGCGT-3′. The cDNA was amplified by an Applied Biosystems (ABI) step-one plus sequence detection system (Applied Biosystems, Foster City, CA). The relative expression was normalized to endogenous controls using the comparative cycle threshold (CT) method, and fold change was calculated as 2^−∆∆Ct^ in gene expression.

### Cell viability assay

For the cell viability assay, 3000 treated cells were seeded into 96-well microtiter plates. The medium was removed and fresh medium was added to each well, after the indicated treatment. After adding 10 µL of CCK-8 solution (CCK-8, Dojin, Japan) into each well, the cells were incubated for 2 h at 37 °C. The absorbance was measured at 450 nm.

### Apoptosis assay

Annexin V-fluorescein isothiocyanate/propidium iodid (Annexin V-FITC/PI) staining was used for the cell apoptosis assay. After treatment, the cells were collected and double stained with Annexin V-FITC/PI according to the manufacturer’s instructions. FACS Calibur flow cytometer (Becton–Dickinson, Franklin Lakes, NJ) was used to analyzed the percentage of apoptotic cells.

### Cell migration assay

A total of 1 × 10^5^ cells/well was added into the upper chamber with 650 μL medium containing 10% FBS in the lower chamber. After 36 h, the nonmigrated cells were removed using a cotton swab and the migrated cells were fixed by 4% formaldehyde, stained with 0.1% crystal violet. The field of view was randomly selected, and the total number of cells was counted.

### Matrigel invasion assay

Matrigel-coated Transwell chambers (BD Biosciences, San Jose, CA) were used for the matrigel invasion assay according to the manufacturer’s instructions. Briefly, 10,000 cells were seeded into the upper matrigel-coated chambers with DMEM containing 10% FBS in the lower chamber. After 36 h, non-invading cells in the upper chamber were removed by scrubbing with a cotton-tipped swab. The invaded cells were fixed with 4% paraformaldehyde and stained with 0.2% crystal violet. The total number of cells in a randomly selected field of view was counted. Six fields from each chamber were photographed.

### Western blot analysis

Cells were homogenized with RIPA-containing buffer [50 mM Tris–Cl pH8.0, 150 mM NaCl, 0.02% NaN_3_, 0.1% SDS, 100 μg/ml phenylmethylsufonyl fluoride (PMSF), 1 μg/ml aprotinin, 1% Triton]. After centrifugation, cell lysates (100 μg/lane) were subjected to 12% SDS-PAGE and transferred onto polyvinylidene difluoride membranes (Millipore). Antibodies against vimentin (Santa Cruz, 1:1000), GAPDH (CST, 1:1000), E-cadherin (CST, 1:1500) and HRP-conjugated goat anti-rabbit secondary antibodies (Promab, 1:1000) were used. Protein bands were detected by the enhanced chemiluminescence reaction and blot film was scanned.

### Cell transfection

siRNA oligonucleotides and negative control were designed from the ~ 350 base pair highly conserved gVC-In4 region within the MALAT1 locus target sequence and synthesized by RiboBio (Co Ltd, China). The miR-142-3p mimic, or control (scrambled negative controls) was designed and synthesized by RiboBio (Co Ltd, China). The SMAD5 full-length sequence was synthesized and subcloned into a pCDNA3.0 vector (Invitrogen, Shanghai, China). Lipofectamine 3000 (Invitrogen) was used for transfection of siRNA, miR-142-3p mimic, mimic control or plasmids into cells following to the manufacturer’s protocol.

#### Luciferase reporter assay

Luciferase reporter plasmids (MALAT1-Wt and MALAT1-mut) were designed and constructed by Generay (Shanghai, China). Two luciferase reporters containing wild-type MALAT1 (psiCHECK2-MALAT1-WT, which encompassed the binding sites for miR-142-3p) or mutant MALAT1 (psiCHECK2- MALAT1-MUT, which encompassed the mutant sequence of the binding sites for miR-142-3p) were constructed. In order to analyze the interaction between MALAT1 and miR-142-3p, the HEK293T cells were co-transfected with luciferase reporter plasmids and miRNA mimics by Lipofectamine™ 3000 transfection. Luciferase reporter plasmids (psiCHECK2-SMAD5-WT and psiCHECK2-SMAD5-MUT) were also designed and constructed by Generay (Shanghai, China). In order to assess the interaction between SMAD5 and miR-142-3p, HEK293T cells were co-transfected with luciferase reporter plasmids and miRNA mimics by Lipofectamine™ 3000 transfection. The dual-luciferase reporter assay system was used for luciferase activity analysis. The relative luciferase activity was calculated by the ratio of firefly luciferase activity to renilla luciferase activity.

### Animal studies

For the xenograft tumor model, the stably over-expressing MALAT1 HepG2 cells and stably knocked down cell line were established by Lv-UBE2CP3, Lv-shMALAT1 and Lv-control viruses. A total of 4 × 10^6^ HepG2 cells were suspended in ice-cold PBS and injected in each nonobese diabetic/severe combined immunodeficiency (NOD/SCID) mouse (5 weeks old, male) to establish xenografts. The formula V =  a × b^2^/2 was used to calculated the tumor volume (a = long axis, b = short axis. All animal experimentation was approved by the Animal Care Committee of Nanfang Hospital, which is affiliated with Southern Medical University.

### EdU assay

The EdU assay was performed as previously described [21]. Briefly, cells were treated with 5-ethynyl-2-deoxyuridine (Click-iT^®^ Plus EdU Alexa-647^®^ Imaging Kit, Life Technologies). Then, cells were washed with phosphate-buffered saline (PBS) and fixed by paraformaldehyde. For image capture, the FV10i confocal microscope (OLYMPUS, Japan) was used, and the EdU + cells in each field were counted.

### Immunofluorescence assays

Immunofluorescence assays were performed as described in our previous study [22]. Antibodies against vimentin (Santa Cruz, 1:500) and E-cadherin (CST, 1:1500) were used and the images were captured using a FV10i confocal microscope (OLYMPUS, Japan).

### Immunocytochemistry

Immunocytochemistry was performed according to the previously described method. Briefly, 4-µm thick paraffin sections were deparaffinized and incubated in 3% hydrogen peroxide to block endogenous peroxidase activity. The slides were blocked from nonspecific antibody binding and incubated with primary antibody against E-cadherin (1:200), N-cadherin (1:200), Ki-67 (1:300) and SMAD5 (1:300). All of the antibodies were purchased by Cell Signaling Technology.

### Statistical analysis

All results are presented as the mean ± standard error of the mean (SEM) from at least three independent experiments. Student’s *t* test was used to assess differences between two groups, and one-way analysis of variance was used for multiple comparisons. A value of P < 0.05 was considered statistically significant.

## Results

### Overexpressed MALAT1 might act as a competing endogenous RNA sponge for miR-142-3p in hepatocellular carcinoma

**Fig. 1:**
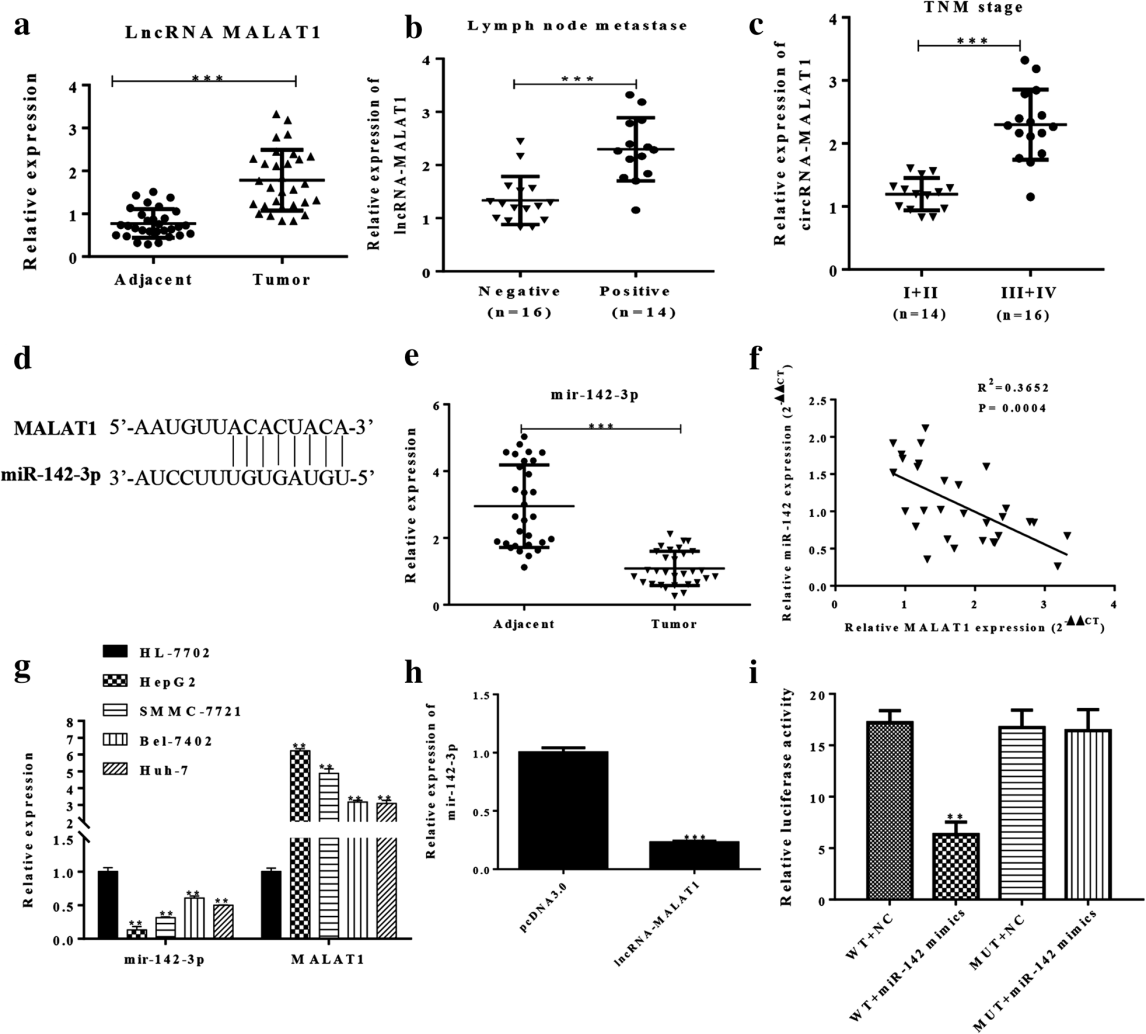
Overexpressed MALAT1 acts as a competing endogenous RNA sponge for miR-142-3p in hepatocellular carcinoma. **a** The expression of MALAT1 in hepatocellular carcinoma tissues and adjacent non-tumor tissues was assessed by Q-PCR. n = 30. **b** The expression of MALAT1 in two subsets tissues (lymph node metastase positive and lymph node metastase negative) was analyzed by Q-PCR. **c** The expression of MALAT1 was significantly overexpressed in cancer subsets (Stage III and Stage IV) with respect to other subsets (Stage I and Stage II). **d** The putative-binding sites between MALAT1 and miR-142-3p were predicted by bioinformatics analysis. **e** Q-PCR was used to analyze the expression of miR-142-3p in hepatocellular carcinoma tissues and adjacent non-tumor tissues. **f** Correlation between relative MALAT1 level and miR-142-3p in hepatocellular carcinoma tissues with linear regression lines. **g** Q-PCR was used to analyze the level of MALAT1 in hepatocellular carcinoma cell lines and a human liver cell line. **h** HepG2 cells were transfected by MALAT1 overexpression plasmid. Q-PCR was used to analyze the levels of miR-142-3p. **i** A luciferase reporter plasmid containing wild-type or mutant MALAT1 was co-transfected with miR-142-3p mimics or NC into HEK-293 T cells. Luciferase assay was performed. Data represent three independent experiments (mean and SEM of triplicate samples). *P < 0.05, **P < 0.01. ***P < 0.001. SEM, standard error of the mean

Firstly, we assessed the relative expression level of MALAT1 in hepatocellular carcinoma tissues and adjacent non-tumor tissues. As shown in Fig. 1a, the expression of MALAT1 was upregulated in hepatocellular carcinoma tissues. The hepatocellular carcinoma tissues were divided into two subsets: lymph node metastase positive and lymph node metastase negative. The level of MALAT1 in hepatocellular carcinoma tissues was significantly higher in lymph node metastase positive subsets than in lymph node metastase negative subsets (Fig. 1b). As shown in Fig. 1c, MALAT1 was significantly overexpressed in cancer subsets (Stage III and Stage IV) with respect to other subsets (Stage I and Stage II). By using the bioinformatics databases (Starbase, RNAhybrid) that predict potential lncRNA-miRNA interactions, we found that miR-142-3p was a putative MALAT1 binding miRNAs (Fig. 1d). Then, we analyzed the expression levels of miR-142-3p in hepatocellular carcinoma tissues and adjacent non-tumor tissues. The results showed that miR-142-3p expression was downregulated in hepatocellular carcinoma tissues compared with adjacent non-tumor tissues (Fig. 1e). Further analysis of hepatocellular carcinoma specimens demonstrated that MALAT1 expression was negatively correlated with the expression of miR-142-3p in corresponding specimens (Fig. 1f, P = 0.0004, R^2^ = 0.3652). Then, we measured the expression levels of MALAT1 and miR-142-3p in hepatocellular carcinoma cell lines and a human liver cell line. Notably, all the hepatocellular carcinoma cell lines—especially the two lines (HepG2, SMMC-7721)—had a higher level of MALAT1 than the human liver cell line. However, all of the hepatocellular carcinoma cell lines had a lower level of miR-142-3p than the human liver cell line (Fig. 1g). Next, the HepG2 and SMMC-7721 cell lines were selected for further study to assess the potential functional role of MALAT1. In HepG2 cells, the MALAT1 was overexpressed and we found that the level of miR-142-3p was downregulated by MALAT1 overexpression (Fig. 1h). Luciferase activity assay was performed to verify the putative-binding sites between MALAT1 and miR-142-3p. The results showed that miR-142-3p downregulated the activity of luciferase reporter harboring wild-type MALAT1 but not the mutant MALAT1 (Fig. 1i). Collective data indicated that MALAT1 might act as a miRNA decoy for miR-142-3p and regulated the expression of miR-142-3p in hepatocellular carcinoma cells.

### Knockdown of MALAT1 inhibited the proliferation and promoted cell apoptosis of hepatocellular carcinoma cells via miR-142-3p

**Fig. 2:**
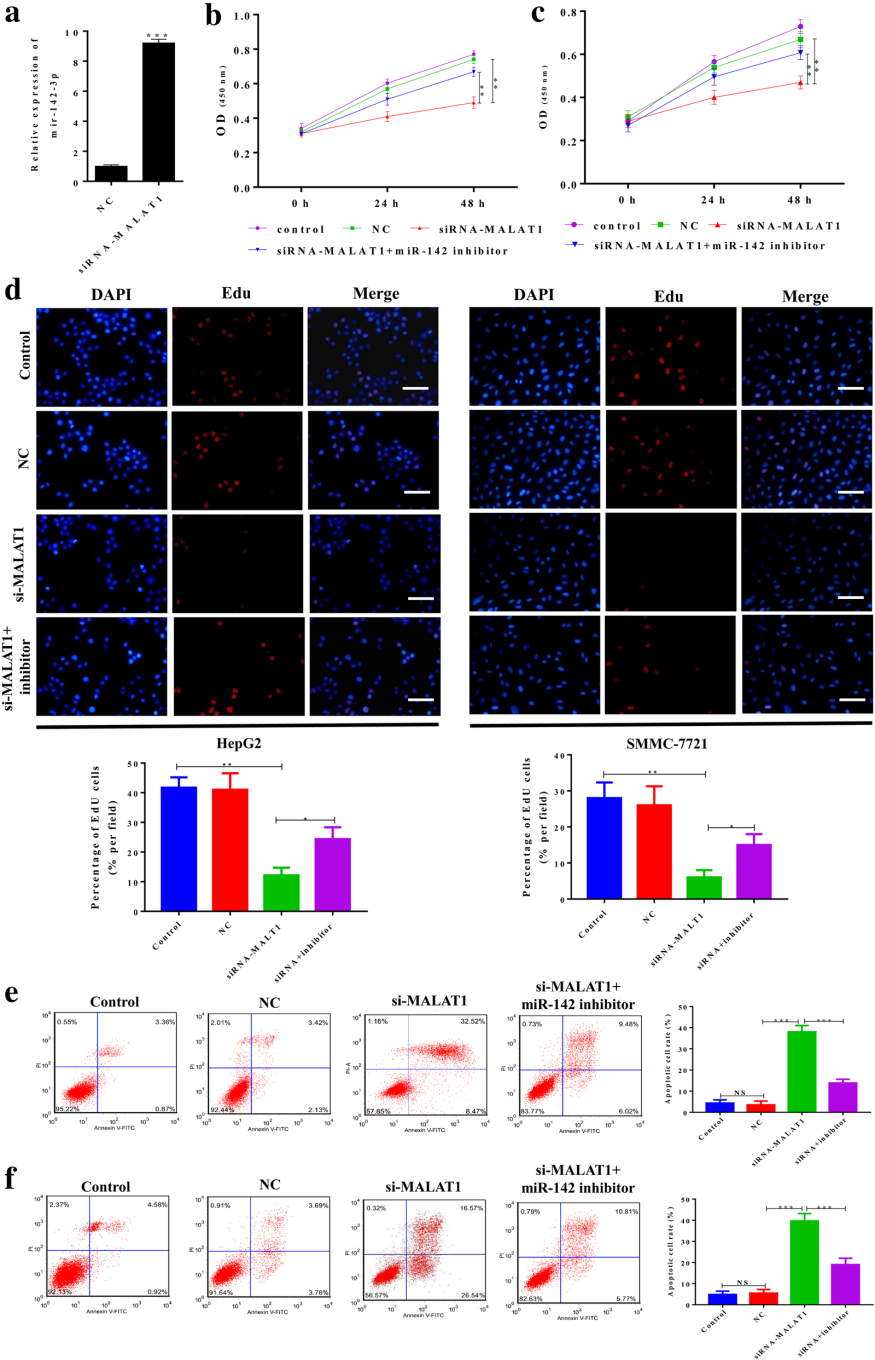
Knockdown of MALAT1 inhibited the proliferation and promoted cell apoptosis of hepatocellular carcinoma cells via miR-142-3p. The expression of MALAT1 in HepG2 and SMMC-7721 cells was knocked down by transfecting with MALAT1 siRNAs or si-control. NC was used as the control. The miR-142-3p inhibitor was used to knock down the level of miR-142-3p. **a** Q-PCR was used to analyze the level of miR-142-3p in HepG2 cells after transfection with MALAT1 siRNAs or control siRNAs (NC). **b**, **c** Cell viability was assessed by CCK-8 assay after the indicated transfection in HepG2 and SMMC-7721 cells. **d** The cell proliferation of HepG2 and SMMC-7721 cells after the indicated transfection was assessed by EdU assay. Representative images of the cells are shown. Scale bar, 100 μm. **e**, **f** Cell apoptosis of HepG2 and SMMC-7721 cells after the indicated transfection was evaluated by flow cytometry. Data represent three independent experiments (mean and SEM of triplicate samples). *P < 0.05, **P < 0.01. ***P < 0.001. SEM, standard error of the mean

Next, we explored the potential role of MALAT1 in hepatocellular carcinoma cells. The expression of MALAT1 was knocked down by siRNA. As shown in Fig. 2a, the expression of miR-142-3p in hepatocellular carcinoma cells was significantly increased by MALAT1 knockdown (Fig. 2a). The expression of MALAT1 and miR-142-3p in HepG2 and SMMC-7721 cells was knocked down alone or together, and cell viability was assessed. As shown in Fig. 2b, c, the knockdown of MALAT1 significantly decreased the cell viability of HepG2 and SMMC-7721 cells, and co-transfection with miR-142-3p inhibitor reversed the effects of knockdown of MALAT1. MALAT1 had a similar effects on cell proliferation as assessed by EdU assay (Fig. 2d). For cell apoptosis, the knockdown of MALAT1 significantly promoted the cell apoptosis of HepG2 and SMMC-7721 cells, while co-transfection with miR-142-3p inhibitor reversed the effects of knockdown of MALAT1 on cell apoptosis (Fig. 2e, f).

### Knockdown of MALAT1 inhibited the migration and invasion of hepatocellular carcinoma cells by EMT

**Fig. 3:**
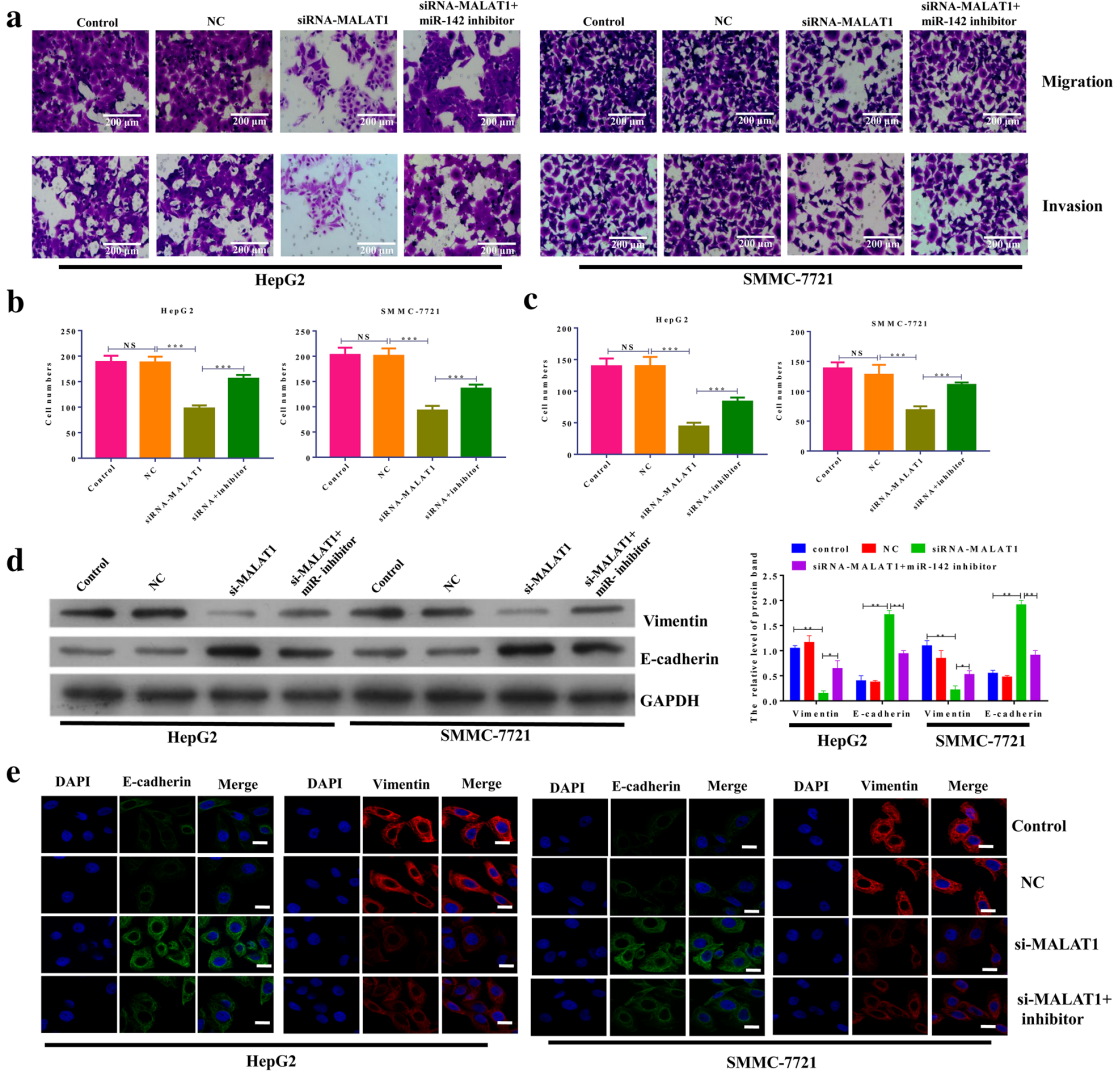
Knockdown of MALAT1 inhibited the migration and invasion of hepatocellular carcinoma cells by EMT. The expression of MALAT1 in HepG2 and SMMC-7721 cells was knocked down by transfecting with MALAT1 siRNAs or si-control. NC was used as control. The miR-142-3p inhibitor was used to knock down the level of miR-142-3p. Magnification, ×200. **a** The cell migration assay and matrigel invasion assay were performed to assess the migration potency of HepG2 and SMMC-7721 cells after the indicated transfection. Representative images of the invaded cells are shown. **b** After the cell migration assay, six fields within each chamber were photographed using an inverted microscope and camera, and migrating cells were counted in each field. The average number is shown. **c** After the matrigel invasion assay, six fields within each chamber were photographed using an inverted microscope and camera, and invading cells were counted in each field. The average number is shown. **d** The protein expression of vimentin and E-cadherin in HepG2 and SMMC-7721 cells after the indicated transfection for 48 h was evaluated by Western blot. The densitometry plot of results from the blots is shown. The relative expression levels were normalized to GAPDH. **e** Immunofluorescence assay assessed the protein levels of vimentin and E-cadherin in HepG2 and SMMC-7721 cells after the indicated transfection. Scale bar, 10 μm. Data represent three independent experiments (mean and SEM of triplicate samples). *P < 0.05, **P < 0.01. ***P < 0.001. Glyceraldehyde 3-phosphate dehydrogenase, GAPDH; EMT, epithelial cell-to-mesenchymal transition; SEM, standard error of the mean

Then, we further ascertained the role of MALAT1 in cell migration and invasion of hepatocellular carcinoma cells. A shown in Fig. 3a–c, the knockdown of MALAT1 significantly inhibited the migration and invasion potency of HepG2 and SMMC-7721 cells. Cotransfection with miR-142-3p inhibitor reversed the effects of knockdown of MALAT1 on migration and invasion potency of HepG2 and SMMC-7721 cells. Analysis of EMT makers by Western blot showed that knockdown of MALAT1 decreased the protein level of vimentin and increased the protein level of E-cadherin in both HepG2 and SMMC-7721 cells. Co-transfection with miR-142-3p inhibitor reversed the effects of MALAT1 knockdown on protein levels of vimentin and E-cadherin (Fig. 3d). Similarly, immunofluorescence assay also confirmed the roles of MALAT1 in regulating the protein levels of vimentin and E-cadherin (Fig. 3e).

### MiR-142-3p suppressed cell proliferation and promoted cell apoptosis by targeting SMAD5 in hepatocellular carcinoma

**Fig. 4:**
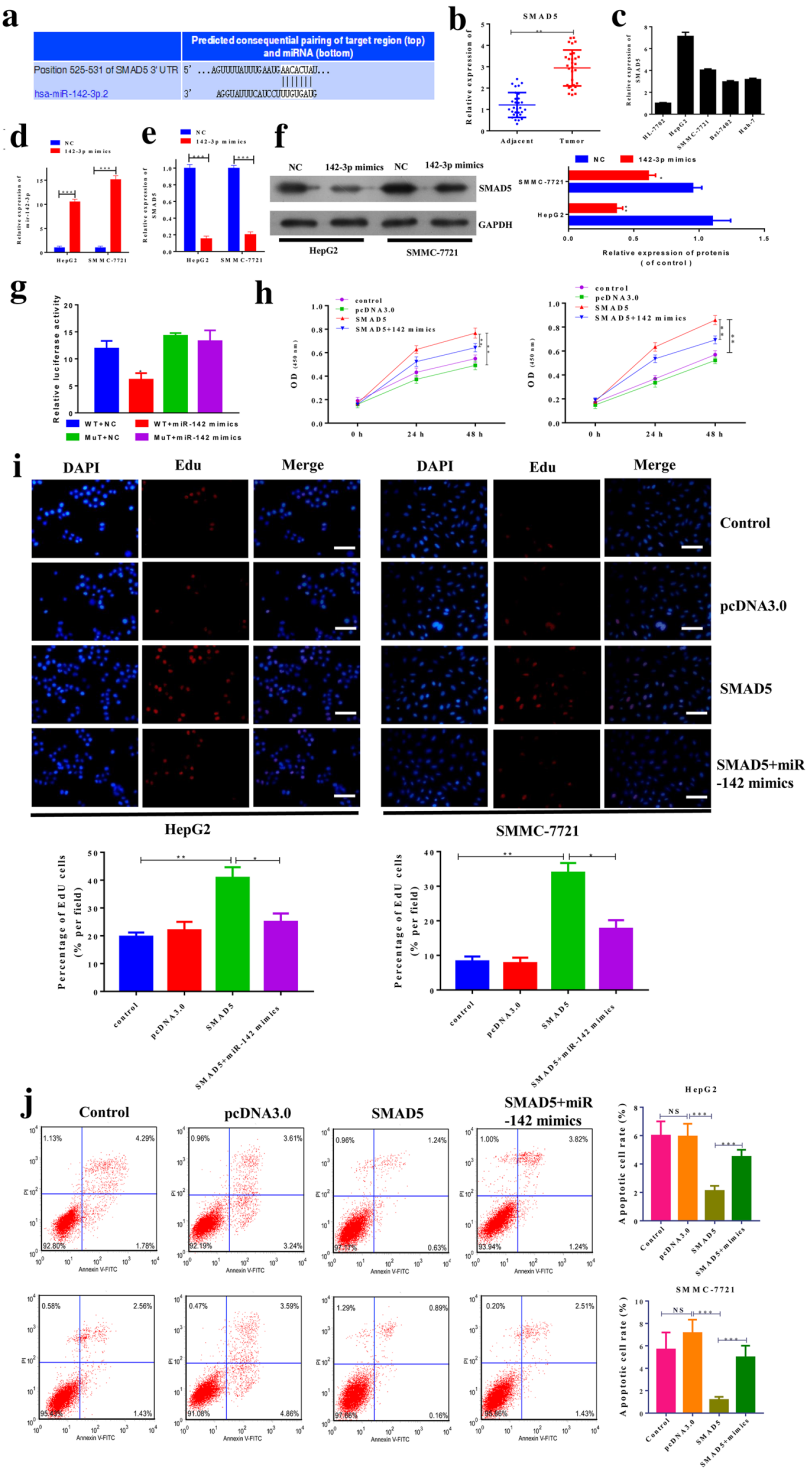
MiR-142-3p suppressed the cell proliferation and promoted cell apoptosis by targeting SMAD5 in hepatocellular carcinoma. **a** Schematic diagrams of the putative interactions between miR-142-3p and SMAD5. **b** The expression of SMAD5 in hepatocellular carcinoma tissues and adjacent non-tumor tissues was assessed by Q-PCR. **c** Q-PCR was used to analyze the mRNA level of SMAD5 in 5 hepatocellular carcinoma cell lines. **d** HepG2 cells were transfected by miR-142-3p mimic control mimic (NC), and the level of miR-142-3p was analyzed by Q-PCR. **e** The HepG2 and SMMC-7721 cells were transfected by miR-142-3p mimic control mimic (NC); the mRNA was analyzed by Q-PCR. **f** HepG2 and SMMC-7721 cells were transfected by miR-142-3p mimic control mimic (NC). The protein level of SMAD5 was analyzed by Western blot. **g** The luciferase reporter plasmid containing wild-type or mutant SMAD5 was co-transfected with miR-142-3p mimics or control mimic into HEK-293 T cells. Luciferase activity was analyzed and normalized to Renilla activity. **h** SMAD5 in HepG2 and SMMC-7721 cells was ectopically expressed using a plasmid containing SMAD5. The plasmid vector pcDNA3.0 was used as the control. The miR-142-3p mimic control mimic (NC) was also used as indicated in the figure. Cell viability was assessed by CCK-8 assay after the indicated transfection in HepG2 and SMMC-7721 cells. **i** The cell proliferation of HepG2 and SMMC-7721 cells after the indicated transfection was assessed by EdU assay. Representative images of the cells are shown. Scale bar, 100 μm. **j** Cell apoptosis of HepG2 and SMMC-7721 cells after the indicated transfection was assessed by flow cytometry. Data represent three independent experiments (mean and SEM of triplicate samples). *P < 0.05, **P < 0.01. ***P < 0.001. SEM, standard error of the mean

To further explore the molecular mechanism of miR-142-3p in regulating cell proliferation, migration and invasion of hepatocellular carcinoma cells, we searched bioinformatics databases. SMAD5 was identified as a putative target of miR-142-3p (Fig. 4a). The expression of SMAD5 in hepatocellular was upregulated in carcinoma tissues compared with adjacent non-tumor tissues (Fig. 4b). The mRNA level of SMAD5 in HepG2 and SMMC-7721 cells was higher compared with three other cell lines (Fig. 4c). In HepG2 and SMMC-7721 cells, miR-142-3p was ectopically expressed by transfection of miR-142-3p mimic, and the transfection efficiency was confirmed (Fig. 4d). As shown in Fig. 4e, the mRNA level of SMAD5 in HepG2 and SMMC-7721 cells was significantly decreased by miR-142-3p ectopic expression. The protein level of SMAD5 in HepG2 and SMMC-7721 cells was also significantly decreased by miR-142-3p ectopic expression (Fig. 4f). Luciferase activity assays confirmed that SMAD5 was a target of miR-142-3p (Fig. 4g).

Next, we explored the roles of miR-142-3p and SMAD5 in HepG2 and SMMC-7721 cells. As shown in Fig. 4h, the ectopic expression of SMAD5 promoted the viability of HepG2 and SMMC-7721 cells, and co-transfection with miR-142-3p mimic reversed the effects of SMAD5 overexpression. Similar results were found for cell proliferation as assessed by EdU assay (Fig. 4i). The ectopic expression of SMAD5 significantly inhibited the cell apoptosis of HepG2 and SMMC-7721 cells, while the co-transfection with miR-142-3p mimic reversed the effects of SMAD5 overexpression in cell apoptosis (Fig. 4j).

### MiR-142-3p suppressed migration, invasion and EMT by targeting SMAD5 in hepatocellular carcinoma

**Fig. 5:**
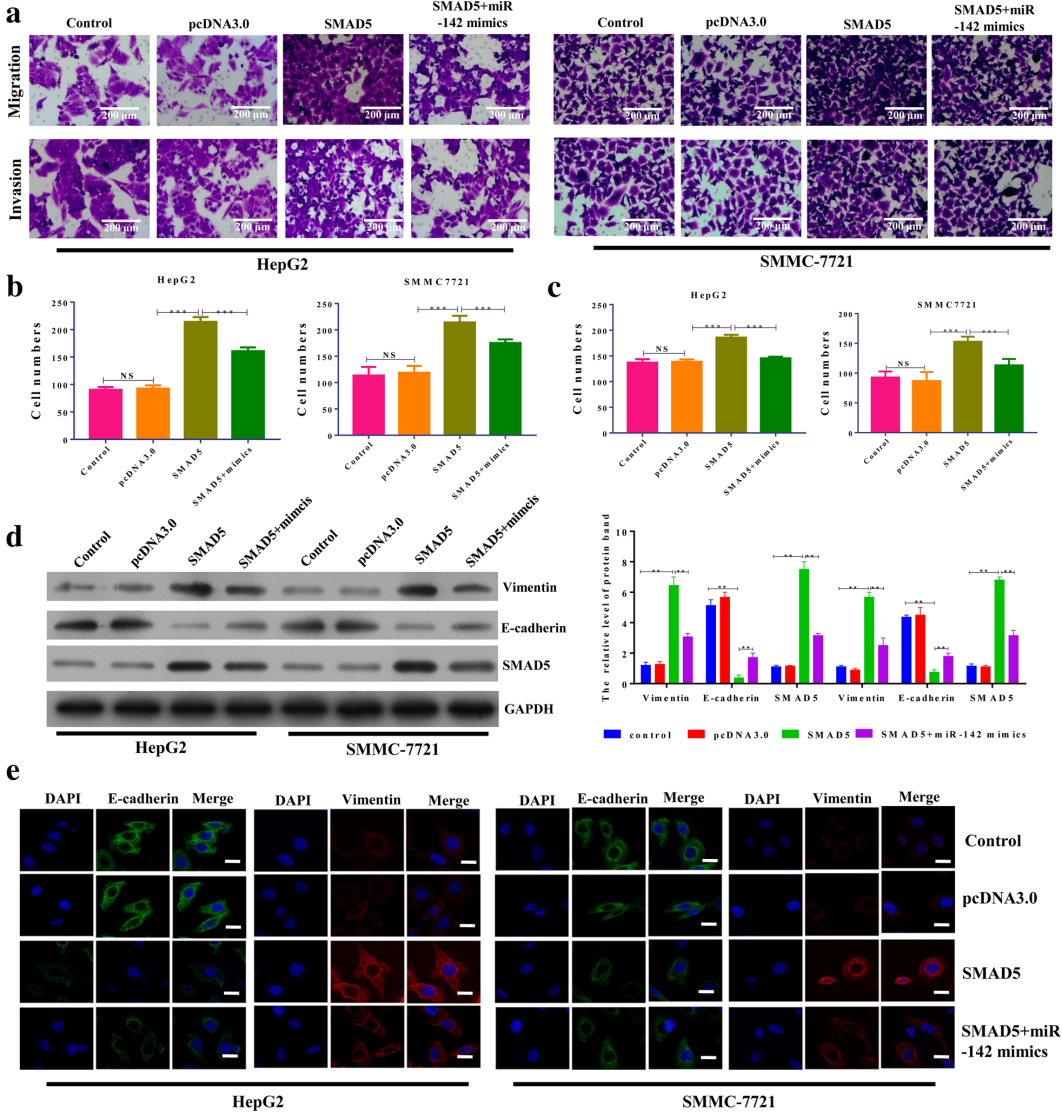
MiR-142-3p suppressed the migration, invasion and EMT by targeting SMAD5 in hepatocellular carcinoma. SMAD5 was ectopically expressed in HepG2 and SMMC-7721 cells by a plasmid containing SMAD5. The plasmid vector pcDNA3.0 was used as control. The miR-142-3p mimic control mimic (NC) was also used as indicated in the figure. **a** The cell migration assay and matrigel invasion assay were performed to assess the migration potency of HepG2 and SMMC-7721 cells after the transfection indicated in the figure. Representative images of the invaded cells are shown. Magnification, ×200. **b** After the cell migration assay, six fields within each chamber were photographed using an inverted microscope and camera, and migrating cells were counted in each field. The average number is shown. **c** After the matrigel invasion assay, six fields within each chamber were photographed using an inverted microscope and camera, and invading cells were counted in each field. The average number is shown. **d** The protein expression of vimentin, SMAD5 and E-cadherin in HepG2 and SMMC-7721 cells after the indicated transfection for 48 h was performed by Western blot. The densitometry plot of results from the blots is shown. The relative expression levels were normalized to GAPDH. **e** Immunofluorescence assay was used to assess the protein levels of vimentin and E-cadherin in HepG2 and SMMC-7721 cells after the indicated transfection. Scale bar, 10 μm. Data represent three independent experiments (mean and SEM of triplicate samples). *P < 0.05, **P < 0.01. ***P < 0.001. Glyceraldehyde 3-phosphate dehydrogenase, GAPDH; EMT, epithelial cell-to-mesenchymal transition; SEM, standard error of the mean

The role of MALAT1 in cell migration and invasion of hepatocellular carcinoma cells was further explored. As shown in Fig. 5a–c, the ectopic expression of SMAD5 significantly increased migration and invasion viability of HepG2 and SMMC-7721 cells. Co-transfection with miR-142-3p mimic reversed the effects of ectopic expression of SMAD5 on migration and invasion viability of HepG2 and SMMC-7721 cells. The ectopic expression of miR-142-3p inhibited the migration and invasion potency of HepG2 and SMMC-7721 cells (Fig. 5a–c). Regarding EMT, Western blot indicated that the overexpression of SMAD5 increased the protein level of vimentin and decreased the level of E-cadherin in both HepG2 and SMMC-7721 cells. Co-transfection with miR-142-3p mimic reversed the effects of ectopic expression of SMAD5 on protein levels of vimentin and E-cadherin (Fig. 5d). Similar results were found by immunofluorescence assay (Fig. 5e). Collectively, miR-142-3p suppressed migration, invasion, and EMT by targeting SMAD5 in hepatocellular carcinoma.

### MALAT1 promoted tumor growth by regulating the expression of miR-142-3p

**Fig. 6:**
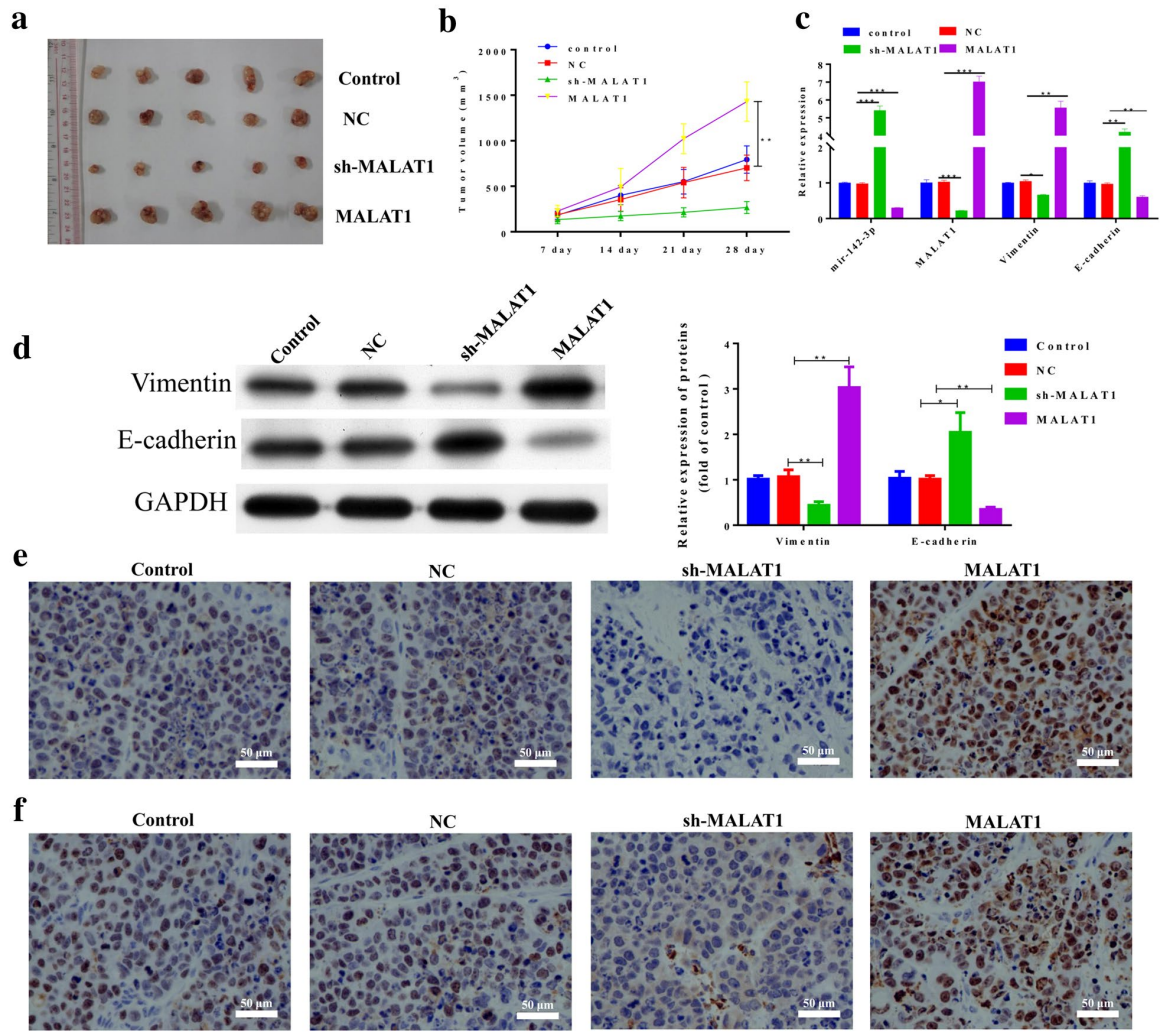
MALAT1 promoted tumor growth by regulating the expression of miR-141-3p and SMAD5. The MALAT1 was stably knocked down and overexpressed in HepG2 cells, and stably cell lines were established. A xenograft model was established by the stably established cell lines. **a**, **b** Representative images of tumors after indicated treatment. The changes in tumor volume were monitored and shown (n = 5 per group). **c** The RNA level of miR-142-3p, vimentin and E-cadherin in the tumor tissues of the indicated group was assessed by Q-PCR. **d** The protein expression of vimentin and E-cadherin in the tumor tissues was evaluated by Western blot. The densitometry plot of results from the blots is shown. The relative expression levels were normalized to GAPDH. **e**, **f** The protein expression of Ki-67 and SMAD5 in the tumor tissues was analyzed by immunohistochemistry. Scale bar, 50 μm. Data represent three independent experiments (mean and SEM of triplicate samples). *P < 0.05, **P < 0.01. ***P < 0.001. Glyceraldehyde 3-phosphate dehydrogenase, GAPDH; SEM, standard error of the mean

MALAT1 was stably knocked down and overexpressed in HepG2 cells to establish stable cell lines. Then, the cell lines were used to establish a xenograft model to investigate the role of MALAT1 in tumor growth. As shown in Fig. 6a, b, the knockdown of MALAT1 significantly inhibited tumor growth, while the ectopic expression of MALAT1 significantly promoted tumor growth. The expression of H19 in tumor tissues of the xenograft models was confirmed (Fig. 6c). Furthermore, the expression of miR-142-3p and E-cadherin was significantly increased in the MALAT1 knockdown group compared with the control group. The expression of miR-142-3p and E-cadherin was significantly decreased in the ectopically expressed MALAT1 group compared with the control group. The MALAT1 knockdown group showed a decrease in expression while the ectopically expressed MALAT1 group showed an increase in expression of vimentin (Fig. 6c, d). In addition, knockdown of MALAT1 decreased the expression of Ki-67 and SMAD5 while ectopic expression of MALAT1 significantly increased the expression of Ki-67 and SMAD5 (Fig. 6e, f).

## Discussion

Previously discovered and widely studied as a lncRNA, MALAT1 has been shown to be involved in the metastasis of early-stage non-small cell lung cancer (NSCLC) and is considered a prognostic marker for stage I of NSCLC [23]. In addition, MALAT1 is associated with other non-cancer diseases, including myocardial infarction and hyperglycemia [18, 24–27]. In the cardiovascular system, MALAT1 was is involved in endothelial cell function and vessel growth [24, 25]. In addition, MALAT1 expression in tissues associated with myocardial infarction is upregulated and plays a vital role in cardiovascular disease [26]. MALAT1 is highly expressed in hepatocellular carcinoma cell lines when compared to normal liver cells [28], and MALAT1-miR-195-EGFR axis was found to play an important role in hepatocellular carcinoma [29]. Our results suggested that the MALAT1 promoted cell proliferation, migration and invasion by the MALAT1-miR-142-3p-SMAD5 axis.

The current study supports findings from other studies that miR-142 is a key regulator of many biological processes and related signaling pathways in embryonic development, homeostasis and disease processes [30]. MiR-142 has been shown to be preferentially expressed in embryonic tissue, hematopoietic tissues, immune cells, tumors tissues, and many other kinds of tissues [31]. In addition, miR-142 has been shown to play important functions in disease, including functional role in cancer, lineage differentiation of hematopoietic cells, virus infection, inflammation, and immune tolerance [30, 32–34]. Regarding cervical cancer, the expression of miR-142-3p is reduced in cervical cancer epithelial cells compared with healthy cervical epithelial cells, suggesting a regulatory role [35, 36]. MicroRNA-142-3p inhibits cell proliferation and invasion of cervical cancer cells by targeting Frizzled class receptor 7 (FZD7). MiR-142-3p had tumor suppressive effects in cell proliferation and invasion in Hela and SiHa cells [36]. Similarly miR-142-3p is involved in tumor progression and invasion in hepatocellular carcinoma cells [37, 38].

In our study, we found that the expression of miR-142-3p was downregulated in hepatocellular carcinoma tissues compared with adjacent non-tumor tissues. MALAT1 acts as a miRNA decoy for miR-142-3p and regulates the expression of miR-142-3p in hepatocellular carcinoma cells. In addition, miR-142-3p suppresses cell proliferation, migration, invasion and EMT, and promotes cell apoptosis by targeting SMAD5 in hepatocellular carcinoma. As an important biological process, EMT is characterized by a progressive loss of epithelial characteristics and acquisition of mesenchymal properties [39]. Collectively, studies have reported that activation of EMT reprogramming plays an important role in tumor progression and drug resistance [40]. In cancer epithelial cancer cells, the activation of EMT is accompanied by downregulated expression of adhesion molecules (E-cadherin) and upregulated expression of mesenchymal proteins (N-cadherin and vimentin) that favor cell motility and invasion [41–43]. Our study showed that the knockdown of MALAT1 inhibited the migration and invasion of hepatocellular carcinoma cells by suppressing EMT. The loss of EMT makers may lead to the inhibition of migration and invasion of hepatocellular carcinoma cells.

In summary, we have characterized the roles of MALAT1 in hepatocellular carcinoma and specifically delineated potential molecular mechanisms. The MALAT1-miR-142-3p-SMAD5 axis plays an important role in cell proliferation, migration, and invasion of hepatocellular carcinoma cells. The findings from the current study have furthered our understanding of hepatocellular carcinoma and identified potential targets for clinical treatment.

## Abbreviations

MALAT1: metastasis-associated lung adenocarcinoma transcript 1
miR-142-3p: microRNA-142-3p
CCK-8: Cell Counting Kit-8
EMT: epithelial cell-to-mesenchymal transition
lncRNAs: long noncoding RNAs
